# Limited Innovations After More Than 65 Years of Immunoglobulin Replacement Therapy: Potential of IgA- and IgM-Enriched Formulations to Prevent Bacterial Respiratory Tract Infections

**DOI:** 10.3389/fimmu.2018.01925

**Published:** 2018-08-23

**Authors:** Jeroen D. Langereis, Michiel van der Flier, Marien I. de Jonge

**Affiliations:** ^1^Section Pediatric Infectious Diseases, Laboratory of Medical Immunology, Radboud Institute for Molecular Life Sciences, Nijmegen, Netherlands; ^2^Radboud Center for Infectious Diseases, Nijmegen, Netherlands; ^3^Pediatric Infectious Diseases and Immunology, Amalia Children's Hospital, Nijmegen, Netherlands; ^4^Expertise Center for Immunodeficiency and Autoinflammation (REIA), Radboudumc, Nijmegen, Netherlands

**Keywords:** IgM, IgA, IgG, immunoglobulin replacement therapy, respiratory tract infections, primary immunodeficiencies, complement system proteins, bacterial infections

## Abstract

Patients with primary immunoglobulin deficiency have lower immunoglobulin levels or decreased immunoglobulin function, which makes these patients more susceptible to bacterial infection. Most prevalent are the selective IgA deficiencies (~1:3,000), followed by common variable immune deficiency (~1:25,000). Agammaglobulinemia is less common (~1:400,000) and is characterized by very low or no immunoglobulin production resulting in a more severe disease phenotype. Therapy for patients with agammaglobulinemia mainly relies on prophylactic antibiotics and the use of IgG replacement therapy, which successfully reduces the frequency of invasive bacterial infections. Currently used immunoglobulin preparations contain only IgG. As a result, concurrent IgA and IgM deficiency persist in a large proportion of agammaglobulinemia patients. Especially patients with IgM deficiency remain at risk for recurrent infections at mucosal surfaces, which includes the respiratory tract. IgA and IgM have multiple functions in the protection against bacterial infections at the mucosal surface. Because of their multimeric structure, both IgA and IgM are able to agglutinate bacteria efficiently. Agglutination allows for entrapment of bacteria in mucus that increases clearance from the respiratory tract. IgA is also important for blocking bacterial adhesion by interfering with bacterial adhesion receptors. IgM in its place is very well capable of activating complement, therefore, it is thought to be important in complement-mediated protection at the mucosal surface. The purpose of this Mini Review is to highlight the latest advances regarding IgA- and IgM-enriched immunoglobulin replacement therapy. We describe the different IgA- and IgM-enriched IgG formulations, their possible modes of action and potential to protect against respiratory tract infections in patients with primary immunoglobulin deficiencies.

## Role for immunoglobulin subclasses in protection against bacterial infections

Immunoglobulin production by B-lymphocytes is a sophisticated adaptive immune defense mechanisms evolved in jawed vertebrates [1]. The absolute importance for immunoglobulins in protection against infections is illustrated in patients that completely lack immunoglobulin production, which is a lethal condition due to increased susceptibility for invasive infection [2]. There are 5 main classes of immunoglobulins; IgG, IgM, IgA, IgD and IgE, all having different properties and functions [3]. For this Mini Review, we will focus on IgG, IgM, and IgA and their role in protection against bacterial respiratory tract infections.

Immunoglobulins are with 10–22 g/L a major serum constituent. The majority of serum immunoglobulins are IgG (~75%), followed by IgA (~15%) and IgM (~10%) [4]. IgG can be subdivided into IgG1, IgG2, IgG3 and IgG4 and IgA can be subdivided into IgA1 and IgA2. The half-life of immunoglobulins vary, with 3 to 4 weeks for IgG1, IgG2 and IgG4, whereas this is around 2 weeks for IgG3 [5, 6]. Half-life for IgA and IgM is with 5 to 6 days much shorter compared to IgG [6, 7].

The different immunoglobulins have specific functions in immunity. Upon binding to their targets, both IgG and IgM are capable of activating complement through binding of C1q. The ability to activate complement for IgG subclasses varies due to differences in C1q binding, which is highest for IgG3, followed by IgG1, IgG2 and is absent for IgG4 [8, 9]. Recent data indicate that IgG oligomerizes into hexamers, forming an optimal configuration for C1q binding [10]. This oligomerization might explain the more efficient activation of complement by IgM, [11–13] because it is present as polymeric structures (pIgM) [14].

IgA is not able to activate complement directly. There are two subclasses of IgA, IgA1 and IgA2, characterized by differences in their hinge region [15]. Whereas IgA1 is the predominant subclass in serum, both IgA1 and IgA2 are present on the mucosal surface [16]. The majority of IgA at the mucosal surface is dimeric (dIgA) [16], whereas the majority in serum is monomeric [17]. IgA is most abundant at mucosal surfaces where it has a role in protection against toxins, viruses and bacteria by means of direct neutralization, or by preventing attachment to the mucosal epithelium [18, 19].

IgA and IgM can be transported to mucosal surfaces through the polymeric immunoglobulin receptor (pIgR) [20]. Only polymeric immunoglobulins containing a J-chain can be transported by this receptor [21]. The pIgR present on the basolateral side of epithelial cells binds dIgA and pIgM, which facilitates transcytosis to the apical side, where it is cleaved off and leaving the secretory components attached, resulting in the formation of secretory IgA (sIgA) or sIgM [22, 23]. Association of the secretory component enhances resistance to proteolysis [24], but does not affect the ability to activate complement [25].

IgG is not transported by the pIgR, but through the neonatal Fc receptor (FcRn) [26]. FcRn is able to transport IgG from the basolateral to the apical side, and vice versa [27]. This FcRn-mediated retrograde transport and recycling of IgG is thought to contribute to the prolonged half-life in serum [28, 29].

A successful strategy to protect healthy individuals from respiratory tract infections is the use of vaccination. In the early 1990s, the *Haemophilus influenzae* serotype B (Hib) conjugate vaccine entered the market, which decreased invasive Hib disease cases substantially in many countries [30–33]. This Hib conjugate vaccine is highly immunogenic and increases capsule-specific antibody levels that not only protects against disease such as pneumonia and meningitis, but also against nasopharyngeal colonization [34]. Hib vaccination elicits a combination of serum immunoglobulin subclasses, mainly IgG, but also IgM and IgA [35]. Vaccination of children with the *Streptococcus pneumoniae* polysaccharide conjugate vaccines (PCV) has shown to increase capsule-specific IgG antibody levels [36] and confer protection against nasopharyngeal colonization, otitis media, pneumoniae and bacteremia [37–39]. Vaccine-induced polysaccharide capsule-specific antibodies initiate complement deposition on the bacterial surface, which is essential for opsonophagocytic killing of *S. pneumoniae* and *H. influenzae* in whole blood [40].

## Immunoglobulin deficiencies

The diverse roles of the different immunoglobulin subclasses in protection against infections is illustrated in patients with primary immune deficiencies (PID), as described in more detail below.

### Selective IgA deficiency

Selective IgA deficiency (sIgAD) is characterized by serum IgA level of <7 mg/dL, with normal levels of both IgG and IgM in individuals more than 4 years of age [41]. Most patients are clinically asymptomatic, but others show recurrent infections, allergies and autoimmunity [2]. Although rather prevalent, the exact pathogenesis is unknown. Since sIgAD is heterogeneous in presentation of disease symptoms, it is likely that different aetiologies might be involved in the cause of this disease. There is evidence for genetic predisposition based on familial clustering, and mutations in many genes involved in cellular and humoral immunity have been associated with sIgAD [42].

Recurrent respiratory tract infections are the most common disease associated with sIgAD [43–45]. When divided into complete and partial IgA deficiency, a history of chronic or recurrent infections is more often found in patients with complete IgA deficiency (77%), compared to partial IgA deficiency (20%) [44], suggesting that a lower IgA is associated with frequent infections. Elevated levels of IgG or IgM are found in a proportion of sIgAD patients [44–47], which might compensate the IgA deficiency. Patients with sIgAD who had higher saliva IgM levels showed a lower infection incidence [47], although this was not found in a later study [46]. Patients with sIgAD are usually not treated with IgG replacement therapy (IgGRT), but it is recommended for individuals with IgA deficiency and concomitant IgG2 subclass deficiency [48].

### Selective IgM deficiency

The European Society for Immunodeficiencies (ESID) Registry defines selective IgM deficiency (sIgMD) as a serum IgM concentration of 2 standard deviations below the normal level, with normal levels of serum IgA and IgG, normal vaccination responses and absence of T cell defects [41]. The immunological and clinical phenotype of sIgMD is very heterogeneous and patients can remain asymptomatic [49]. Similar to sIgAD, patients with sIgMD often present with recurrent respiratory problems [49, 50]. In a cohort of 17 sIgMD patients, recurrent upper respiratory tract infections were observed in 5 out of 6 patients with undetectable IgM levels (<0.05 g/L) [49]. Although for most sIgMD patients IgGRT was not required [49], it is recommended for patients with recurrent or severe infections [51].

### Common variable immune deficiencies

Patients with common variable immune deficiencies (CVID) are characterized by a marked decrease of IgG or IgA with or without low IgM levels, poor specific immunoglobulin responses to vaccination and no profound T-cell deficiency [41]. A monogenic cause has been identified in 2–10% of CVID patients, but most patients appear to be polygenic or multifactorial disorders [52]. Most CVID patients present with infectious manifestations, commonly of the upper and lower respiratory tract [53]. The majority (70%) of CVID patients develop chronic pulmonary complications including bronchiectasis and bronchial wall thickening [54]. To prevent especially respiratory tract infections, IgGRT is the mainstay treatment for CVID patients [55].

### Agammaglobulinemia

Patients with agammaglobulinemia have very low or no serum immunoglobulin levels, making these patients highly susceptible to infections [2]. The largest group of patients have X-linked agammaglobulinemia (XLA), which is a caused by a defect in the *Btk* gene encoding Bruton Tyrosine Kinsase (Btk), which accounts for 85% of agammaglobulinemia patients. IgGRT is recommended for all agammaglobulinemia patients to reduce infections [56]. Bacterial infections of the respiratory tract are often seen in patients with agammaglobulinemia, prior to diagnosis, but also after initiation of IgGRT [57–60], likely due to the absence of IgA and IgM antibodies in IgGRT. As a results, a large proportion of agammaglobulinemia patients develop chronic lung diseases [57, 59].

## Past, present and future of immunoglobulin replacement therapy

IgG for IgGRT is traditionally collected from the Cohn fraction II after cold methanol precipitation [61, 62] and used to prevent infections in patients with PID. Despite IgGRT, recurrent infections, mainly of the respiratory tract, are reported [57–60], and is associated with a lowered life-expectancy of CVID and agammaglobulinemia patients [63, 64]. Currently used immunoglobulin preparations contain only IgG. As a result, concurrent IgA and IgM deficiency persists in a large proportion of immunoglobulin deficient patients, which results in recurrent infections and development of chronic lung diseases such as bronchiectasis [65, 66]. Here, we summarize the current and novel immunoglobulin replacement therapies possibly effective in preventing bacterial respiratory tract infections.

### In the beginning; 1952

IgGRT was first introduced in 1952 by Colonel Ogden Bruton and applied to the first patient with agammaglobulinemia [67]. This patient had an extensive history of infections, including osteomyelitis, gastrointestinal infection, and multiple episodes of epidemic parotitis, otitis media, pneumonia and sepsis for 4.5 years. After the third episode of epidemic parotitis, it was found that the serum of this patient was deficient for immunoglobulins. The patient was given subcutaneous IgGRT (SC-IgGRT) and was free of invasive infections [67, 68]. After this first success, more individuals with agammaglobulinemia and other PID were treated with IgGRT. Most patients received intramuscular IgGRT (IM-IgGRT) [60] because uptake and bioavailability of IgG from the muscle is better as compared to fatty tissue where it is deposited by SC-IgGRT [69]. However, injections were painful and the volume that can be given is limited, therefore, alternative administration methods were explored.

An advantage for SC-IgGRT is the ability for home treatment, which was shown to be feasible and safe [70]. Home treatment is more convenient, and prevents loss of school or workdays and hospital costs [70]. Although SC-IgGRT was initially limited by a slow infusion, this improved considerably over the past years [71]. Recently, a new SC-IgGRT formulation containing recombinant hyaluronidase, which is an enzyme that cleaves the extracellular matrix and increases tissue permeability. Therefore, the use of recombinant hyaluronidase enables administration of larger volumes of IgG and thereby decreases dosing frequency to once in the 3–4 weeks, is in clinical trial [72].

### Next step, intravenous IgG replacement therapy

The largest disadvantage of both SC-IgGRT and IM-IgGRT is the limited volume that can be administered per dose. As a result, the patient requires frequent dosing, typically every week. With intravenous IgGRT (IV-IgGRT), larger volumes can be giving, which decreases the frequency of dosing to once in the 3–4 weeks. However, it requires venous access and the incidence of systemic adverse reactions were high in the early years, mainly due to vasomotor and cardiovascular manifestations, which was most likely due to immunoglobulin aggregation and complement activation [56, 73, 74]. Later, modification of IV-IgGRT by for instance ß-propiolactone, which prevents aggregation [75], reduced the number of side-effects considerably [76] and soon became the routine treatment regimen [77, 78]. In a head-to-head comparison, IV-IgGRT was given once every 4 weeks and SC-IgGRT every week. Significant improvement in clinical parameters such as a lower number of days with acute respiratory infections and IgG trough levels were found for IV-IgGRT [74]. This even improved further when IV-IgGRT was given once every 3 weeks [74]. Important for protection against respiratory infections, serum opsonic capacity of *H. influenzae* and *S. pneumoniae* increased when IV-IgGRT was administered once every 3 weeks as compared to once every 4 weeks [74].

## Possible improvements; IgA- and/or IgM-enriched immunoglobulin replacement therapy

Nowadays, both SC-IgGRT and IV-IgGRT are used to prevent infections in patients with PID and are proven to be safe [79]. The choice of therapy is mainly based on the clinical condition of the patient, side effects and patient's preference, as SC-IgGRT therapy can be administered by the patient at home independent of help from health care professionals. However, as mentioned above, the current IgGRT has an important limitation; it contains only IgG, resulting in the absence or low levels of IgA and/or IgM in patients with PID. Despite IgGRT, many patients with PID develop chronic lung disease [57, 59, 66]. Therefore, improvements in therapy are needed, which might be the addition of IgA and/or IgM.

There are a limited number of IgA- and/or IgM-enriched immunoglobulin preparations used in clinical practice. Here, we summarize studies that have used IgA- and/or IgM-enriched immunoglobulin products, mainly in treatment of acute bacterial infections in general and highlight the studies focused on infections in immunoglobulin deficient patients.

### Fresh frozen plasma

Fresh frozen plasma (FFP) was advocated over IM-IgGRT by Richard Stiehm in 1975 [80] and was next to IM-IgGRT used in the early days [81]. The IgG levels achieved with FFP treatment were significantly higher as compared to IM-IgGRT, while IgM and IgA levels were only slightly increased [80]. The use of FFP has limited or no side effects, but to reach a normal level of IgA and IgM, it should be given at a high frequency (~ twice a week), which is inconvenient for the patient. FFP was also given to PID patients with chronic gastro-intestinal infections as add-on therapy to their normal IgGRT. Two patients with relapsing *Campylobacter jejuni* infection were given FFP for 2 or 4 weeks (500 mL twice weekly), which resulted in complete recovery of both patients [82]. Plasma infusion resulted in detectable serum IgA levels in two patients and detectable serum IgM levels in 1 patients [82]. Although we have not found direct evidence, FFP might be beneficial as an treatment, next to normal IgGRT, to eliminate chronic bacterial respiratory tract infections.

### Pentaglobin

Pentaglobin is an IgA- and IgM-containing immunoglobulin preparation collected from Cohn fraction III, consisting of 72% IgG, 12% IgM, and 16% IgA for intravenous use [83]. Pentaglobin is treated with ß-propiolactone, which decreases immunoglobulin aggregation, but also affects complement fixation and Fc-binding capacity [84, 85]. A clear beneficial effect of Pentoglobin in comparison to conventional intravenous IgG is its effective reduction of endotoxin [86, 87]. Direct effects on bacterial killing has also been observed by *in vitro* experiments. For instance, Pentaglobin had a greater opsonic activity against *Pseudomonas aeruginosa, Staphylococcus aureus* and *Escherichia coli*, in comparison to IV-IgGRT [88, 89]. In a *S. aureus* mouse sepsis model, the number of bacteria in liver and kidney were significantly lower for animals receiving Pentaglobin in comparison to animals receiving IV-IgGRT Intratect [89]. Pentaglobin has to our knowledge not been used as replacement for conventional IgGRT, but, it has successfully been applied to treat two hypogammaglobulinemia patients with persistent gastroenteric *C. jejuni* infections [90]. In these two patients, six dosages of Pentaglobin at 3-week intervals were well tolerated [90]. After the first dose, an increased serum bactericidal antibody activity against *C. jejuni* was measured and treatment resulted in the elimination of the *C. jejuni* infections [90]. This result is in line with the use of IgA- and IgM-containing FFP, which was also successfully used to treat chronic *C. jejuni* infections [82]. We have found a remarkable increase in complement-mediated killing of *C. jejuni* [31] by supplementing purified serum IgM to serum of a patients with agammaglobulinemia, supporting an important role for IgM in complement-mediated protection against bacterial pathogens.

### Trimodulin

Trimodulin (BT-588, predecessor BT-086) contains twice the amount of IgA (21%) and IgM (23%) in comparison to Pentaglobin. Trimodulin is not treated with ß-propiolactone and showed approximately 10-fold increase in opsonization of *E. coli* compared to Pentaglobin [91]. In a rabbit model for endotoxemia, Trimodulin showed an increased elimination of *E. coli* from the bloodstream [92]. Recently, the results of a phase II trial, which included 160 patients with severe community-acquired pneumonia, were published [93]. Although no difference was observed for ventilator-free days between the placebo and Trimodulin groups, *post-hoc* analyses supported improved outcome in patients with elevated CRP, reduced IgM, or both. In addition, Trimodulin seemed to prevent secondary bacterial infections because infection-related treatment-emergent adverse events were significantly decreased from 57% in the placebo group to 33% in the Trimodulin-treated patients [93]. Since IgA and IgM concentrations present in Trimodulin are higher compared to Pentaglobin, we would expect an additive effect on preventing bacterial infections of the respiratory tract, although this has not been determined to date.

### IgAbulin

IgAbulin is an IgA-enriched IgG preparation, which was shown to prevent necrotizing enterocolitis when administered orally to babies with low birth weight [94] and has successfully been used to treat children with chronic diarrhea [95]. Oral administration of IgAbulin daily for 3 weeks decreased the number of stools per day and improved the consistency of the stools [95]. So far, IgA-enriched IgGRT has not been used to prevent or treat bacterial respiratory tract infections. Since IgA is the most abundant immunoglobulin isotype on the respiratory tract mucosal surface, with divers functions in preventing bacterial infections, it might help in preventing and clearing bacterial infections. A possible limitation of the current IgA-enriched immunoglobulin preparations is that they are isolated from plasma, and plasma-derived IgA is mainly IgA1, which is more sensitive to bacterial IgA proteases [96], in comparison to IgA2, which is most abundant on the mucosal surface [16]. In addition, the majority of serum-derived IgA is monomeric [17], in contrast to locally produced IgA, which is mainly dimeric [16]. But, administration of sufficient IgA might potentially be effective in preventing bacterial respiratory tract infections, especially in PID patients with concurrent IgA deficiency.

### Purified serum IgA and IgM linked to recombinant secretory component

Although not in clinical trials, plasma-derived IgA and IgM containing recombinant secretory component have been tested in animal studies with promising results. It has been shown that plasma-derived IgA and IgM can bind recombinant secretory component to form sIgA and sIgM [97]. Addition of the secretory component increased resistance to protease activity and showed to prevent *Shigella flexneri*-induced intestine epithelial cell damage in an *in vitro* cell model [98]. sIgA and sIgM preparations were found to bind and agglutinate *Salmonella enterica* Typhimurium [99]. Oral administration of sIgA and sIgM preparations effectively limited *S. Typhimurium* infection and systemic dissemination in mice [99]. No results were published regarding the effects on protection of bacterial respiratory pathogens, but since these sIgA and sIgM preparations are able to block bacterial adhesion, it is expected to prevent acquisition of bacteria in the respiratory tract when applied to the airways. In addition, these sIgA and sIgM preparations are not treated with ß-propiolactone, preserving natural complement fixating activity. Future experiments are required to address the efficacy to prevent bacterial respiratory tract infections.

## Concluding remarks

Current IgGRT treatment has not shown to prevent bacterial respiratory tract infections in a selection of PID patients. Based on the clinical presentation of patients with IgA and IgM deficiencies, who mainly present with respiratory tract infections, it is conceivable that IgA- and/or IgM-enriched immunoglobulin replacement therapy with biologically active IgA and/or IgM have the potential to prevent these type of infections. In the near future, pre-clinical *in vitro* assays, animal experiments and clinical trials have to be conducted to determine whether IgA and/or IgM-enriched immunoglobulin replacement therapy would prevent bacterial respiratory tract infections in patients with PID.

## Author contributions

JL wrote the initial manuscript. MvdF and MdJ edited and approved the final manuscript.

### Conflict of interest statement

The authors declare that the research was conducted in the absence of any commercial or financial relationships that could be construed as a potential conflict of interest.

## Abbreviations

Btk: Bruton tyrosine kinase
CVID: Common variable immune deficiency
dIgA: Dimeric IgA
FcRN: Neonatal Fc receptor
FFP: Fresh frozen plasma
IgGRT: IgG replacement therapy
IgRT: Immunoglobulin replacement therapy=
IM-IgGRT: Intramuscular IgG replacement therapy
IV-IgGRT: Intravenous IgG replacement therapy
PID: Primary immune deficiencies
pIgM: Polymeric IgM
SC-IgGRT: Subcutaneously IgG replacement therapy
sIgA: Secretory IgA
sIgAD: Selective IgA deficienciy
sIgM: Secretory IgM
sIgMD: Selective IgM deficienciy
XLA: X-lined agammaglobulinemia.
